# Variations in DREB1A and VP1.1 Genes Show Association with Salt Tolerance Traits in Wild Tomato (*Solanum pimpinellifolium*)

**DOI:** 10.1371/journal.pone.0132535

**Published:** 2015-07-10

**Authors:** Eguru Sreenivasa Rao, Palchamy Kadirvel, Rachael C. Symonds, Subramaniam Geethanjali, Ramadihalli N. Thontadarya, Andreas W. Ebert

**Affiliations:** 1 Division of Vegetable Crops, ICAR-Indian Institute of Horticultural Research, Bengaluru, India; 2 Crop Improvement Section, ICAR-Indian Institute of Oilseeds Research, Rajendranagar, Hyderabad, India; 3 School of Biosciences, The University of Nottingham Malaysia Campus, Jalan Broga, Semenyih, Selangor, Darul Ehsan, Malaysia; 4 Department of Millets, Centre for Plant Breeding and Genetics, Tamil Nadu Agricultural University, Coimbatore, India; 5 Genetic Resources and Seed Unit, AVRDC–The World Vegetable Center, Shanhua, Tainan, Taiwan; Louisiana State University Agricultural Center, UNITED STATES

## Abstract

Association analysis was conducted in a core collection of 94 genotypes of *Solanum pimpinellifolium* to identify variations linked to salt tolerance traits (physiological and yield traits under salt stress) in four candidate genes viz., DREB1A, VP1.1, NHX1, and TIP. The candidate gene analysis covered a concatenated length of 4594 bp per individual and identified five SNP/Indels in DREB1A and VP1.1 genes explaining 17.0% to 25.8% phenotypic variation for various salt tolerance traits. Out of these five alleles, one at 297 bp in DREB1A had in-frame deletion of 6 bp (CTGCAT) or 12 bp (CTGCATCTGCAT), resulting in two alleles, viz., *SpDREB1A_297_6* and *SpDREB1A_297_12*. These alleles individually or as haplotypes accounted for maximum phenotypic variance of about 25% for various salt tolerance traits. Design of markers for selection of the favorable alleles/haplotypes will hasten marker-assisted introgression of salt tolerance from *S*. *pimpinellifolium* into cultivated tomato.

## Introduction

Soil salinity can be due to high concentration of the ions K^+^, Na^+^, Ca^2+^, Mg^2+^ and Cl^-^ [1]. When the electrical conductivity (EC) of the saturation extract in the root zone is in the range of ≥4 dS m^-1^, which is equivalent to 40 mM NaCl, soils are considered saline [2]. Cultivated tomato is moderately sensitive to salinity and tolerates up to 2.5 dS m^-1^ EC. Above this threshold, the crop suffers at least a 10% yield decrease for each unit of increase of soil salinity [3]. Plants react to the presence of increased levels of salt in the soil solution in two phases: a rapid osmotic phase in which increased levels of salt in the soil result in increased osmotic stress to the roots causing a reduction in leaf and shoot growth, and a second ion-specific phase, which starts to affect plant growth when salt accumulates to toxic concentrations in the older leaves [4]. Osmotic stress, ion imbalance and toxicity reduce nutrient uptake and transport capacity leading to nutritional imbalances [5], and finally necrosis and premature death of older leaves.

Three mechanisms of salinity tolerance in plants are recognized: The first is osmotic tolerance which is regulated by long distance signals and occurs prior to Na^+^ accumulation in the shoots. The second mechanism is ion exclusion, in which Na^+^ and Cl^-^ ion accumulation in the shoots is minimized. This is a glycophytic response that restricts the entry and accumulation of the potentially toxic ions Na^+^ and/or Cl^-^ into the shoots. The third mechanism is tissue tolerance, which is the ability of the plant to compartmentalize the toxic ions Na^+^ and/or Cl^-^ into vacuoles, to keep the enzymatic reactions in the cytosol and/or organelles functional [6]. In this context, the maintenance of a high cytosolic K^+^/Na^+^ ratio and the precise regulation of ion transport are critical for plants to tolerate salinity stress.

Plant tolerance to salinity stress involves the functions of many genes that control a range of biochemical and physiological processes. In recent years, substantial research has focused on genetic engineering of candidate genes for abiotic stress tolerance in model plants under controlled conditions, and this has resulted in insights on the role of these genes in key physiological and biochemical processes [7]. Over 18,000 patents have been granted that invoke plant salinity tolerance [1]. Genes that have been used in genetic engineering to improve abiotic stress tolerance include those that facilitate the accumulation of organic compounds with low molecular weight, which serve as osmolytes and contribute to osmotic adjustment. Overexpression of different vacuolar antiports facilitates the exclusion of toxic ions from the cell cytosol [8], while enhanced expression of detoxification enzymes reduces oxidative stress [9]. Other candidate genes that encode regulatory and signaling proteins include, for example, dehydration-responsive element binding (DREB) proteins [10], protein kinases [11], and aquaporins [12]. A comprehensive review of the genes and regulatory networks involved in the improvement of tolerance to drought and salinity stresses has been published [13].

Linkage mapping has been useful to identify major QTLs conferring salt tolerance in plants [14–16] and to exploit them in breeding programs [17]. However, salt tolerance is genetically complex involving diverse mechanisms and governed by multiple genes and often strongly impacted by the environment resulting in limited progress. In this regard, association or linkage disequilibrium (LD) mapping complements traditional mapping efforts for precision and power of identifying causal variants. This strategy originally established in human and animal genetics has successfully been extended to plants during the last decade [18, 19], and recently in tomato [20–22]. Association mapping is performed in two ways: genome wide association study (GWAS), which surveys genetic variation in the whole genome to find signals of association for target traits [23] and candidate gene based association study (CGAS), which relates to linking phenotypic variation with the polymorphic sites in candidate genes to identify the causative polymorphisms [24].

Population structure and multiple testing are two issues that need to be addressed while conducting association analysis. The population structure in the genotype panel may lead to false positive associations if the frequency of a certain phenotype varies across sub-populations. The structure issue has been effectively addressed by the development of a statistical model, called unified mixed linear model (MLM) [25], which takes into account both population structure (Q) and relative kinship (k) information while performing association analysis. Multiple testing refers to an instance that involves the simultaneous testing of several hypotheses and if the multiplicity of tests is not taken into account, the probability that some of the true null hypotheses are rejected by chance alone may be unduly large. False discovery rate (FDR) correction of observed P values has been suggested by Storey and Tibshirani [26] to address this issue.

While genetic variability for salt tolerance traits is limited in cultivated tomato, sources of tolerance have been reported among wild *Solanum* species: *S*. *pimpinellifolium*, *S*. *peruvianum*, *S*. *cheesmaniae*, *S*. *habrochaites*, *S*. *chmielewskii* and *S*. *pennellii* [27]. Introgression of salt tolerance traits from distant wild relatives to cultivated *S*. *lycopersicum* is difficult due to crossing barriers and linkage drag. Finding sources of salt tolerance in *S*. *pimpinellifolium* will be helpful as it is the closest wild relative and readily crossable with *S*. *lycopersicum*. *S*. *pimpinellifolium* also has been a source of useful genes for many other important horticultural traits of cultivated tomato including yield and disease resistance [2]. It has been suggested that *S*. *pimpinellifolium* accessions could be the most promising sources for improvement of salt tolerance in tomato cultivars [28, 29]. This species is native to the Andean region of Peru and Ecuador in Western South America [30], where it is found in dense populations located in undisturbed areas and adapted to diverse environmental conditions ranging from the coastal desert climate to humid and foggy conditions of higher altitudes [31]. Considering that the natural range of *S*. *pimpinellifolium* encompasses environments as diverse as the Ecuadorian tropical forest and the coastal Peruvian desert, this species is a potential source of beneficial alleles for abiotic stress tolerance.

For the present study, we focused on four major genes that have a demonstrated contribution to salinity stress tolerance; NHX1 [32], VP1.1 [33], tonoplast intrinsic proteins (TIP), [12] and dehydration-responsive-element-binding (DREB) protein gene DREB1A [34]. The gene NHX1 is a member of the intracellular NHX antiporter family which catalyzes Na^+^, K^+^/H^+^ exchange and is associated with diverse growth and development processes [35]. It is essential for active K^+^ uptake into the vacuole for regulation of turgor and stomatal function [36]. The overexpression of AtNHX1 has conferred improved salt tolerance in Arabidopsis [37] and tomato [32] with salinity tolerance being correlated with increased levels of NHX1 expression. Higher levels of NHX1 expression were reported in *S*. *pimpinellifolium* compared to *S*. *lycopersicum*, both in the absence of salt stress and after subsequent exposure to salt stress confirming the importance of NHX1 in salt tolerance determination [38]. The NHX1 gene was originally described as a vacuolar Na^+^/H^+^ antiporter [37], however recent evidence supports a role for NHX1 in the subcellular partitioning of K^+^ rather than Na^+^ [39]. Tomato plants overexpressing AtNHX1 accumulated large K^+^ vacuolar pools but showed no increase in Na^+^ accumulation with salinity stress [39]. Plants overexpressing AtNHX1 exhibited increased tolerance to salinity stress which was postulated to be derived from the role that AtNHX1 plays in K^+^ homeostasis. The AVP1 gene encodes for a vacuolar H^+^ pyrophosphatase located to the tonoplast and plasma membrane [33]. Arabidopsis plants overexpressing AVP1 show increased tolerance to salinity, presumably as a result of increased levels of ion uptake into the vacuole [40]. Salinity tolerance was further increased in transgenic tomato by the co-overexpression of AVP1 and AtNHX1 genes [41]. The TIPs, members of the aquaporin family, targeted to the vacuolar membrane have recently been characterized in tomato [12]. Ectopic expression of tomato SlTIP2;2 in both tomato [42] and Arabidopsis [43] produced plants with salinity stress tolerance which was suggested to be a result of a lower osmotic potential and higher water content under salinity stress. The stress inducible transcription factors DREBs, have been studied extensively in response to environmental stress [44]. They are transcriptionally up regulated in response to abiotic stresses such as drought, salinity and cold, and ectopic expression has been associated with improved salinity stress tolerance in Arabidopsis [44], tobacco [45], and potato [46]. It has not yet, to our knowledge, been investigated in relation to salinity stress tolerance in tomato, however it shows potential as a key single gene that is able to confer tolerance to multiple abiotic stresses when upregulated. The DREB family of transcription factors, of which DREB1A is a member, have been suggested to confer increased salinity tolerance through its involvement in signal transduction pathways for the elevated expression of stress responsive genes such as LEA genes and sugar biosynthesis [45].

Recent work of our group has focused on constructing a core germplasm collection of *S*. *pimpinellifolium* [47] and phenotypic expression of salinity tolerance traits in *S*. *pimpinellifolium* in comparison with cultivated tomato [48]. In this study, our objectives were to: (a) characterize four candidate genes (DREB1A, VP1.1, NHX1, and TIP), reportedly involved in salt tolerance, for nucleotide diversity and LD in a *S*. *pimpinellifolium* core germplasm panel; (b) determine the association between candidate gene polymorphisms and phenotypic traits selected for salt tolerance in the germplasm panel; and (c) identify favorable haplotypes in the candidate genes associated with high salt tolerance in *S*. *pimpinellifolium* accessions.

## Results

### Phenotypic evaluation

Salt tolerance was assessed both as *per se* performance under salt stress and relative performance under salt stress compared with control conditions for various yield and survival traits according to the definition of Munns and James [49].

A wide variation with near normal distribution was observed in the germplasm panel for various survival and yield traits under both stress and non-stress conditions (S1 Table). Analysis of variance (ANOVA) revealed significant differences (P = 0.01) among the genotypes for all traits in both stress and non-stress conditions.

Most of the traits recorded skewness values less than 1 and kurtosis values less than 3 suggesting near normal distribution except traits like Na content, K/Na ratio under salt stress and most yield related traits under both control conditions and salt stress, which recorded moderate skewness (S1 Table). Interestingly, most of these yield related traits were also correlated with population structure suggesting the effect of genetic background for the observed deviation from normality. Lack of fertilization during the experiment may be another plausible reason for positive skewness in all the yield related traits.

**Fig 1:**
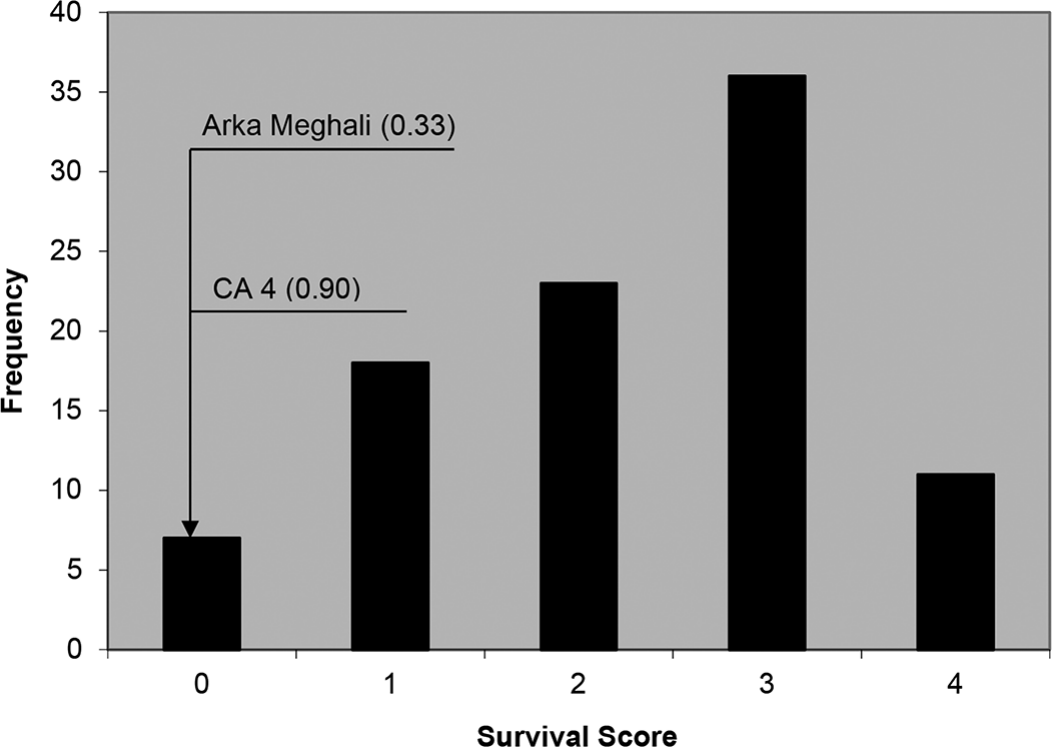
Distribution of survival score ratings among 94 accessions of *S*. *pimpinellifolium* under salt stress along with *S*. *lycopersicum* checks ‘CA4’ and ‘Arka Meghali’.

Compared to control conditions, significant reduction under salt stress was observed for chlorophyll content (t-value = 24.2), plant height (t-value = 21.8), shoot dry weight (t-value = 26.7), fruit set percentage (t-value = 4.1), fruit number (t-value = 7.4), and yield per plant (t-value = 10.8) at 1% significance and average fruit fresh weight (t-value = 2.4) at 5% significance. The survival ratings among the 94 accessions were distributed normally ranging from 0 (low) to 4 (high) with an average of 2.0. The control accessions possessed a low survival score of 0.3 for ‘Arka Meghali’ and 0.9 for ‘CA4’ (Fig 1). Further details of the phenotyping results including phenotypic correlation coefficients among major agronomic traits and a list of selected genotypes with high survival score, with high fruit number/yield and two genotypes combining high survival score and high fruit number are presented in a previous paper [48].

### Population structure and kinship

A total of 204 SSR markers were used to understand the population structure in the panel of 94 accessions of *S*. *pimpinellifolium* employing a model-based approach as implemented in STRUCTURE. Fifty data sets were obtained by setting the number of possible clusters (K) from 1 to 10 with five replications each. The results were then permuted for each K value using CLUMPP software. Applying the second-order statistics (∆K) developed by Evanno et al. [50], there was a sharp peak of ∆K at K = 2, suggesting two major populations.

**Fig 2:**
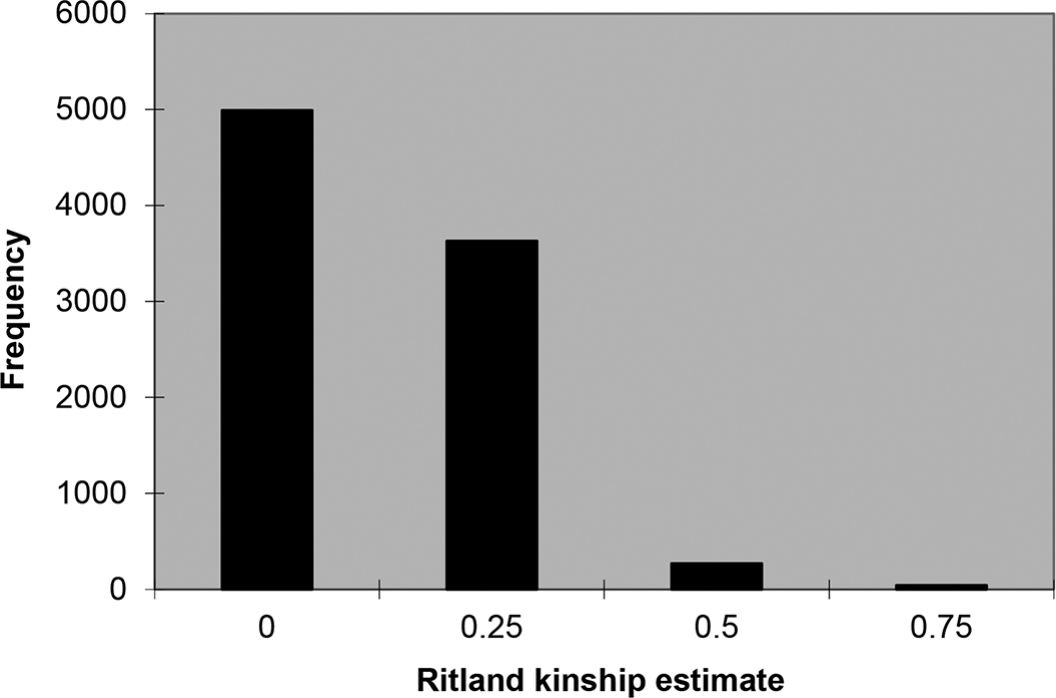
Distribution of pairwise relative kinship estimates among 94 *S*. *pimpinellifolium* accessions. Values are from SPAGeDi estimates using 204 SSR markers.

Using the kinship matrix, which was developed based on 204 SSR markers, relative kinship estimates revealed that 55.4% of the pairwise kinship estimates were equal to 0 whilst 96.4% were below 0.3, with a continuously decreasing number of pairs falling in higher estimated categories (Fig 2).

### Sequence diversity and intragenic LD for candidate genes

The candidate gene sequence analysis covered a concatenated length of 4594 bp and a total of 33 SNPs and 11 indels with allele frequency greater than 5% were detected in the panel of 94 genotypes (Table 1). This is equal to one SNP/indel per 107 bp. Large differences were found in the number of SNPs/indels and nucleotide diversity parameters across the four candidate genes. The SNP/indel frequency varied from 1/87 bp (NHX1) to 1/131 bp (TIP) and the number of SNPs/indels varied from 18 in the VP1.1 gene to 4 in the TIP gene. The total nucleotide diversity ranged from 0.0031 for NHX1 and VP1.1 to 0.0023 for TIP genes (Table 1).

**Table 1:** Summary of parameters of nucleotide variability in the four candidate genes DREB1A, VP1.1, NHX1, and TIP for salt tolerance.

Gene	Samples	Length (bp)	Indels (MAF*>0.05)	SNPs (MAF>0.05)	SNP/Indel frequency	PiPerBP	ThetaPerBP	TajimaD
DREB1A	69	1418	5	9	1/101.00	0.00204	0.0027	-0.6845
VP1.1	72	1521	2	11	1/117.00	0.0019	0.0031	-1.1643
NHX1	73	1130	2	11	1/86.92	0.00386	0.0031	0.7356
TIP	85	525	2	2	1/131.25	0.0015	0.0023	-0.7811

*MAF: Minimum Allele Frequency

The LD was calculated for each candidate gene using TASSEL 2.1. Out of 255 locus-pair comparisons in the four candidate genes, 91 pairs (35.68%) had significant R^2^ value above the generally accepted threshold of 0.1 (Table 2). Seven pairs were in complete LD. However, TIP, VP1.1 and DREB1A recorded a lower percentage of pairs in LD compared to NHX1 (Table 2).

**Table 2:** LD analysis of the four candidate genes DREB1A, VP1.1, NHX1, and TIP for salt tolerance.

	No of pairwise comparisons	No of significant pairwise comparisons (R^2^>0.1)	% significant pairwise comparisons	SNPs in complete LD
DREB1A	92	17	18.47	1
VP1.1	79	19	24.05	2
NHX1	78	55	70.51	4
TIP	6	0	0	0

### Marker trait associations

STRUCTURE analysis indicated that the germplasm panel had two major populations. Therefore, it was of interest to know if the traits evaluated in this study showed correlation with population structure. It was noted that most of the traits did not correlate with the population structure except chlorophyll content under control, plant height under salt stress, fruit number and average fruit weight under control, and salt stress conditions (Table 3).

**Table 3:** Trait correlation (R^2^) with population structure (Q) under control conditions, salt stress and percent reduction due to stress.

Trait	Control	Salt stress	% reduction due to stress
Chlorophyll content	3.226**	2.019	0.066
Na content	-	0.353	-
K content	-	1.336	-
K/Na ratio	-	1.93	-
Survival score	-	2.248	-
Plant height	2.071	3.971**	1.935
Shoot dry weight	2.065	1.942	1.36
Fruit set	1.079	0.983	0.589
Fruit number	3.581**	3.585**	0.717
Fruit yield	1.934	0.571	0.099
Average fruit weight	7.472**	7.867**	1.069

** significant at P = 0.01

Mixed linear model based association analysis recorded 28 marker trait associations across the four candidate genes at a significance level of 0.01 (Table 4). A total of seven traits for DREB1A, four traits for VP1.1, three traits for NHX1 and one trait for TIP have been found to be associated in at least one of the environments (control and/or salt stress). DREB1A and NHX1 recorded significant associations especially for yield related traits both under control and salt stress conditions while the physiological traits were associated only under salt stress. VP1.1 did not record any associations under control conditions and all the trait associations were only under salt stress.

**Table 4:** Traits associated (P</ = 0.01) with various sites of four salt tolerance candidate genes under control conditions and salt stress and trait change due to stress conditions.

Trait		P value		
	SNP/Indel position	Control	Salt stress	% reduction due to stress
**DREB1A**				
Fruit number	13	3.63E-04	6.23E-04	-
	1058	0.0013	-	-
	900	0.0041	-	-
Fruit set	13	0.0085	-	-
Fruit yield	1058	0.0015	0.0066	-
	900	0.0041	-	-
K/Na ratio	297	-	3.96E-05	-
K content	15	-	0.009	-
Plant height	297	-	-	6.51E-04
Survival score	900	-	0.007	-
**VP1.1**				
Chlorophyll content	470	-	7.28E-06	0.0027
	1191	-	7.28E-06	0.0027
	983	-	1.41E-04	0.0036
	1446	-	0.0029	-
	1481	-	0.0029	-
Fruit set	1	-	0.0071	-
K/Na ratio	1	-	0.0035	-
Plant height	587	-	0.0033	-
**NHX1**				
Chlorophyll content	776	0.0039	0.0013	-
Fruit yield	948	0.0061	-	-
Fruit number	948	-	0.0079	-
**TIP**				
Average fruit weight	23	-	-	0.006

After the P values were corrected for false discovery rate (FDR), only five alleles showed significant associations (Table 5). Among them, two were in DREB1A (13 bp and 297 bp positions) and three in VP1.1 (470 bp, 983 bp and 1191 bp positions) (Table 5). None of the variations in NHX1 and TIP showed significant association with salt tolerance traits after FDR correction.

**Table 5:** Traits associated with SNP’s of candidate genes at FDR (q value) </ = 0.05.

Gene	SNP/Indel position bp	Trait	P value	q value	R^2^ value
**DREB1A**	13	Fruit number under control conditions	3.63E-04	5.00E-02	0.1774
	13	Fruit number under salt stress	6.23E-04	5.00E-02	0.1695
	297	K/Na ratio under salt stress	3.96E-05	1.25E-02	0.2577
	297	Plant height percent reduction due to salt stress	6.51E-04	5.00E-02	0.2002
**VP1.1**	470	Chlorophyll content under salt stress	7.28E-06	9.33E-04	0.2407
	983	Chlorophyll content under salt stress	1.41E-04	1.20E-02	0.2219
	1191	Chlorophyll content under salt stress	7.28E-06	9.33E-04	0.2407

The DREB1A variant at 13 bp position (D*REB1A_13*) was found to be associated with fruit number under both control (R^2^ = 17.7%) and salt stress conditions (R^2^ = 17.0%). Two variants of DREB1A were found at 297 bp position: 6 bp (CATCTG) (*DREB1A_297_6)* or 12 bp (CATCTGCATCTG) (*DERB1A_297_12)* deletions, which showed the most significant association with salt tolerance traits viz., K/Na ratio under salt stress (R^2^ = 25.7%) and plant height reduction under salt stress (R^2^ = 20.0%).

Variations at 3 positions in VP1.1 (470 bp, 983 bp and 1191 bp) showed significant association with chlorophyll content under salt stress explaining an almost equal phenotypic contribution (R^2^ = 22.2% to 24.1%).

### Haplotype analysis

Intragenic haplotypes for variations possessing significant associations with salt tolerance traits were manually derived for DREB1A and VP1.1 genes. These haplotypes were scored as multiallelic genotypes as suggested by Barendse [51]. The matrix of this genotypic data was used for association analysis to determine the effects of haplotypes on various salt tolerance traits.

Three haplotypes with minor allele frequency (MAF) >0.05 were found in DREB1A and two haplotypes were found in VP1.1. The haplotype definitions for DREB1A are Hap1: 0_13_−0_297_ (n = 21); Hap2: 0_13_−12_297_ (n = 35); and Hap3: 1_13_−12_297_ (n = 8) [where subscripts refer to the nucleotide positions; 0, 1 (T) and 12 (CATCTGCATCTG) refer to the number of nucleotides deleted; n refers to sample size]. The haplotype definitions for VP1.1 are Hap1: G_470_-A_983_-A_1191_ (n = 63) and Hap2: A_470_-T_983_-C_1191_ (n = 8). Haplotypic associations were observed for DREB1A and VP1.1 with the same traits for which individual associations were found. The phenotypic contributions of haplotypes were also similar to those of individual associations (R^2^ = 17.9% to 24.4%) (Table 6).

**Table 6:** Traits associated with haplotypes of DREB1A and VP1.1 genes at FDR (q value) </ = 0.05.

Gene	Trait	P value	q value	R^2^ value
**DREB1A**	K/Na ratio under salt stress	0.00025	0.0036	0.2444
	Plant height percent reduction due to salt stress	0.00200	0.0194	0.2002
	Fruit number under control conditions	0.00340	0.0247	0.1873
	Fruit number under salt stress	0.01200	0.0498	0.1785
**VP1.1**	Chlorophyll content under salt stress	0.00014	0.0036	0.2107

### Effect of trait associated variations on protein sequence

**Fig 3:**
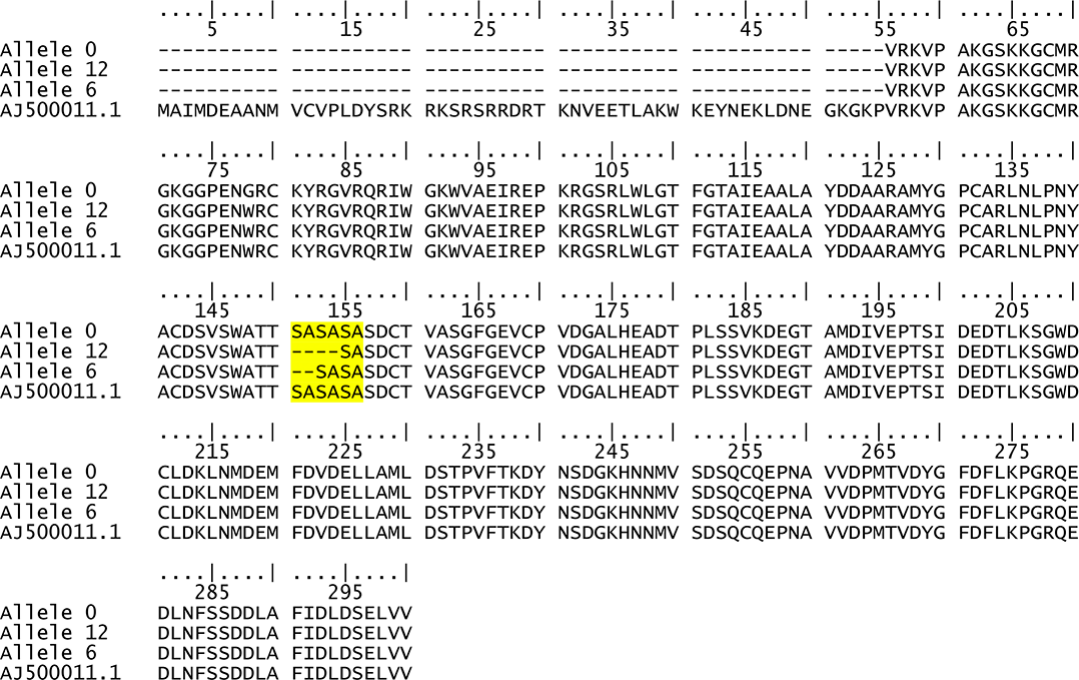
Predicted protein sequence alignment for alleles corresponding to 297 bp position in DREB1A gene identified in a panel of 94 accessions of *S*. *pimpinellifolium* along with a reference sequence of DREB1A in *S*. *lycopersicum* (AF 500011.1). The amino acid sequence variation corresponding to 297 bp is highlighted.

All the five allelic variations were observed in exonic regions. The variant DREB1A_13 had a frame shift mutation because of a 1 bp deletion. The deletion of nucleotide T at 13 bp position led to a change from proline in wild type (allele 0) to leucine (allele 1) and resulted in premature termination of coding sequence. The variant DREB1A_297 had in-frame deletions of 6 bp or 12 bp at 297 bp position. The 6 bp (CATCTG) and 12 bp (CATCTGCATCTG) deletions at this position led to a change in amino acid sequence from SASASA (allele 0) in wild type to SASA (allele 6) and SA (allele 12), respectively (Fig 3). Since these alleles possessed the most significant effect on salt tolerance in *S*. *pimpinellifolium*, we name the alleles 6 and 12 as *SpDREB1A_297_6* and *SpDREB1A_297_12* respectively. Three alleles of VP1.1 had synonymous variations, which did not cause any change in the protein sequence.

## Discussion

A suitable association mapping panel should encompass as much phenotypic and molecular diversity as can be reliably measured in a common environment [52]. Breseghello and Sorrells [53] have reviewed the choice of populations for association analysis in plant breeding programs and suggested that core collections representing the genetic diversity of a species available in genebanks are attractive for association analysis due to the presence of abundant allele diversity. Core collections are useful materials for association analysis of adaptive traits like stress tolerance because of wide allele diversity [54]. The process of selection of a minimum sample with maximum variation while developing a core set has a normalizing effect that is expected to reduce population structure and LD between unlinked loci, thus creating a situation favorable for association analysis [53]. A core set of *S*. *pimpinellifolium* was therefore developed [47] for this purpose and is fairly represented in the current experiments. A total of 27 out of the 94 *S*. *pimpinellifolium* genotypes (S2 Table) originated from sites close to the sea and might have naturally adapted to high salinity conditions.

### Population structure and kinship

The accuracy of marker-trait association is affected by the relatedness among the members of the germplasm panel in which marker effects are estimated. Gowda et al. [55] demonstrated that close relatedness may lead to a substantial increase in the proportion of total genotypic variance explained by the identified QTL resulting in over-optimistic judgment of the precision of marker-assisted selection. The two subpopulations identified in our analysis of population structure indicated distinct subdivisions based on their center of origin.

In this study we used 204 SSR markers to estimate kinship, which is considerably higher than the 100 SSR markers recommended by Yu et al. [56]; hence, a robust kinship estimate can be expected. The kinship analysis indicated that 96% of the pairwise kinship estimates were below 0.25, suggesting that most lines had no or weak relationship with the other lines in this panel—which was to be expected, as the genotypes used in this study belong to a recently established *S*. *pimpinellifolium* core collection [47].

### Sequence diversity and intragenic LD

The Tajima’s D test [57] for all four candidate genes showed no significant difference between π and θ, indicating much lower selection pressure, as would be expected for a wild species. Abundant diversity and rapid LD decay in a germplasm panel are ideal for candidate gene association mapping. The genes TIP, VP1.1 and DREB1A recorded a lower percentage of pairs in LD compared to NHX1 (Table 2). The diverse LD decay rates for different candidate genes indicate a biased and non-uniform evolution of individual genes within the same species as reported by Yu et al. [56].

### Marker trait associations

The current germplasm panel appeared not to be skewed for salt tolerance in terms of population structure. Yu and Buckler [58] warned that the presence of population structure within association mapping panel may often generate spurious genotype-phenotype associations. The spurious associations cannot be controlled completely by population structure alone as the Q matrix gives only a rough dissection of population differentiation. Therefore, Yu et al. [25] suggested incorporating the pairwise kinship into the mixed model to correct for relatedness in association mapping. Hence, we employed a mixed linear model approach that accounts for both population structure (Q) and relative kinship (k) as implemented in the TASSEL 2.1 software package [59]. The P values were corrected for false discovery rate to address the problem of multiple testing.

We considered the five putative allelic variants as major alleles as they explained larger phenotypic variation (17.0% to 25.8%). Edae et al. [60] suggested that a QTL that explains more than 10% of phenotypic variation can be considered a major locus in association mapping. Furthermore, Yu et al. [61] reported that the power and precision of detecting a causal factor in a small panel size of around 100 individuals will be high only for genetic factors accounting for >10% of the phenotypic variation.

Among four candidate genes studied in this experiment, DREB1A and VP1.1 showed association with physiological traits; (DREB1A with K/Na ratio and plant height percent reduction under salt stress; VP1.1 with chlorophyll content under salt stress) supporting their role in adaptation to salt stress. However, only DREB1A had association with yield traits (fruit number) under both control and stress conditions. Elevated levels of DREB1 expression have been associated with adaptation to a wide range of abiotic stresses such as heat, drought, cold, and salinity [44].

Several studies have revealed the reduction in chlorophyll content as a response to salt stress. High shoot Cl^-^ content under salt stress was inversely correlated with leaf chlorophyll content [62, 63]. This observation strongly supports the association of VP1.1 with chlorophyll content under salt stress found in this study.

The results also indicated significant associations with survival traits under stress but not yield traits except in the case of DREB1A_13 which showed association with fruit number irrespective of the stress condition. The possible pleiotropic effect of DREB1A on yield traits needs to be studied in detail. Although survival traits have a low correlation with fruit yield under salt stress [48], a full understanding of the mechanism of these traits would assist breeders in the development of a tomato ideotype for high yield under stress and non-stress conditions.

### Haplotype analysis

In association analysis, every putative marker usually would have multiple alleles in the mapping panel and each of those alleles would contribute differently to the associated trait [64]. Finding a right combination of highly effective alleles at multiple loci within the candidate gene that forms a tolerant haplotype would be more relevant than the individual allele itself at a single locus. It is not sufficient to consider only the effects of an individual SNP when it is possible that another SNP is always modified at the same time. For instance, Jia et al. [64] reported that haplotypes may be advantageous over individual SNPs in the presence of multiple disease susceptibility alleles, particularly when LD between SNPs is weak. The importance of haplotyping is better realized when the actual mutation causing trait variation is not linked with any one SNP but linked with a specific haplotype, and the haplotype effect is stronger than individual SNPs. Therefore, it was in our interest to analyze the association of DREB1A and VP1.1 haplotypes with salt tolerance traits. In the current study, common trait associations of individual SNPs/indels and haplotypes could be detected for K/Na ratio under salt stress, plant height percent reduction and fruit number (DREB1A), and chlorophyll content under salt stress (VP1.1). However, the haplotypes did not improve the phenotypic performance, suggesting that the haplotype effect is only due to LD and not functional synergism among the candidate variations. Hence candidate SNP selection would be as effective as the haplotype selection in the current scenario.

### Effect of trait associated variations on protein sequence

SNPs exist throughout the entire genome, within and outside of coding regions. SNPs residing outside coding regions can occur in intergenic sequences, 5’- or 3’-untranslated regions, intronic regions, and associated non-coding regions such as promoter and transcription factor binding sites. The five alleles exhibiting major associations (R^2^>10%) with salt tolerance traits were of exonic origin, of which DREB1A alleles at 13 bp and 297 bp positions caused amino acid sequence change due to frame shift change and in-frame deletions, respectively. The VP1.1 alleles had synonymous mutations without altering the amino acid sequence. Two hypotheses can explain their significance, the most likely one being that these SNPs do not correspond to the functional mutation but are in LD with it. All markers in strong LD with a functional mutation will appear as significant in association tests. Another possible explanation is that these synonymous SNPs do not lead to amino acid residue changes at protein level; however, they are documented as leading to changes in mRNA structure, stability and splicing or even in delay or acceleration of protein folding that can result in different final protein conformations and functionality [65].

Genes from the DREB family have been isolated and characterized in a number of plants including Arabidopsis [66], wheat [67, 68], rice [69, 70], and maize [71, 72]. Transgenic tomatoes containing a CBF1 (DREB1B) transcription factor from Arabidopsis had enhanced tolerance to water deficit stress, but were also stunted [73]. Growth retardation could be reversed with exogenous application of the growth hormone gibberellic acid. Plant dwarfism and abnormal phenotype are two major aspects that currently limit the practical use of DREB genes of Arabidopsis in crop breeding through a transgenic approach [34]. However, the alleles, *SpDREB1A_297_12* (allele 12) and *SpDREB1A_297_6* (allele 6) or the haplotypes found in the current experiments can be a good alternative for traditional tomato breeding, without adverse effects like dwarfing and other abnormal phenotypes.

The allele *SpDREB1A_297_12* which possessed the highest R^2^ value in the current experiment recorded a lower reduction in plant height (0.38), more number of fruits (41.08), better survival score (2.10) and a lower K/Na ratio (2.08) compared to the wild type allele (0.41; 13.07; 1.73 and 2.39 respectively). This indicates a favorable effect of this DREB1A allele through tissue tolerance rather than ion exclusion. Further studies are required to validate this mechanism.

Generally, once genes that determine the genetic basis of a trait are known, developing functional markers to select for favorable alleles is an important aspect of using genetic information in practical plant breeding [61]. A functional marker is a marker developed from a SNP/indel within a gene that is responsible for variation in the trait [74]. The use of functional markers in molecular plant breeding is more advantageous than linked markers because the latter may lose their diagnostic capacity in breeding populations due to recombination between the marker and the causative SNP region in subsequent generations [74]. Since the functional markers are developed from SNPs within a gene, molecular information can be used confidently across breeding programs to select promising alleles for a trait of interest [75]. The information generated in this experiment can be used to develop functional markers for pyramiding of desirable alleles of multiple genes of interest through marker-assisted backcrossing. However, the benefit of the favorable alleles detected in wild tomato has to be validated in elite lines.

The future experiments may also consider the following possibilities: firstly, the small germplasm panel used in this study might be useful for detection and analysis of major QTLs, but a larger panel would be more effective for investigating the role of minor loci in stress tolerance. Secondly, the marker-trait associations that were found in this study could further be demonstrated in linkage mapping using bi-parental populations to rule out the possibility of false positives as the covariance between genotypes and phenotypes can be broken by generating controlled crosses [76]. Combining association analysis with linkage mapping would be helpful, especially for validating rare variants. Thirdly, only three accessions [#26, #46 and #72; S1 Table] recorded the highest survival score of 4.0 [48] in spite of several accessions possessing favorable alleles in the candidate genes studied. This observation indicates that other important genes are likely involved in the expression of salt tolerance. Therefore a genome-wide association study could probably provide information on more key genes implicated with salt tolerance in *S*. *pimpinellifolium*.

## Materials and Methods

### Plant materials and experimental design for salt stress

AVRDC—The World Vegetable Center (AVRDC) maintains a collection of 330 accessions of *S*. *pimpinellifolium* [77]. From this collection, a subset of 94 accessions was chosen for the study based on passport data, genetic structure, and a fair representation of the core set developed earlier [47]. Details of the 94 *S*. *pimpinellifolium* accessions and their country of origin as well as the collection site, where available, are provided in S2 Table. A total of 27 accessions originated from coastal areas. Two cultivated tomato genotypes—the Indian cultivar ‘Arka Meghali’ and an inbred line ‘CA4’, frequently used in AVRDC’s tomato breeding program were included as checks.

The trials were conducted in net houses at AVRDC headquarters, Taiwan with mean day and night temperatures of 25°/16°C and a photoperiod of 15 hours. We used a split-plot design with two treatments consisting of salt stress (200 mM NaCl) and standard irrigation water (control). Three replications were used per treatment with 3 plants each. The plants were raised in seedling trays with peat moss and were transplanted after 5 weeks into 20 L plastic pots filled with a sterilized mixture of peat, decomposed organic residues, and soil in the ratio of 1:1:1 which possessed an EC of 1 dSm^-1^ measured by the saturated paste method [78]. A progressive salinization technique was employed for imposition of salt stress. All pots in the salt treatment were irrigated with 500 ml of 200 mM NaCl solution every alternate day, while pots of the control were irrigated with 500 ml of normal irrigation water (0 mM NaCl) at the same frequency. The salinity treatment level was based on prior studies that helped establish a clear distinction between salt tolerance and susceptibility of *S*. *pimpinellifolium*, both in terms of survival and yield related traits. No fertilizer was applied in this experiment to avoid interference with the salt treatment. Stress imposition started from the ninth week of sowing, when most of the genotypes started flowering. The duration of the stress treatment was 11 weeks. The average EC of the substrate at the end of the experiment in the salt treatment was 40 dSm^-1^, while the substrate of the control measured 1 dSm^-1^.

### Phenotypic evaluation and data analysis

During the 11 weeks of stress imposition, various physiological traits as well as yield and yield components were recorded under both treatments. Leaf chlorophyll content was measured at each fourth expanded leaf from the plant top at 15 weeks after sowing using a portable Minolta Chlorophyll Meter SPAD 500. Similarly, leaf sodium and potassium concentrations were measured at each fourth expanded leaf from the plant top at 15 weeks from sowing using Cardy sodium and potassium ion meters (HORIBA, Model C-122 and C-131, Japan). The selected leaves were crushed and homogenized in a sterilized polycover using a metal ball roller. One hundred microliters of homogenized sap was used for estimating the sodium and potassium concentrations.

Plant height (cm) was recorded from the base of the plant to the tip of the meristem and shoot dry weight (g) including all above ground parts except fruits was recorded at the end of the experiment. Dry weight was determined after drying at 80°C in an oven until a consistent weight was recorded. A survival score (0–4 scale) was given based on the mode of the performance of a group of plants under stress in each replication for all accessions including check varieties. Percent fruit set was calculated by dividing the total number of fruits by the total number of flowers set on 3-7^th^ trusses, multiplied by 100. Number of fruits per plant was determined from four harvests at breaker stage. Average fruit weight (g) was obtained by dividing total fruit fresh weight by the total number of fruits collected from a single plant. Yield per plant (g) was determined by measuring total fruit weight.

The performance of 94 genotypes and two check varieties for the above traits, except leaf sodium and potassium concentrations (due to negligible concentrations in the control) and survival score under salt stress was compared with their performance under control conditions to obtain percent change in performance due to salt stress. The mean data of the above traits were used to calculate broad-sense heritability (H) based on between and within accession variances. The statistical analysis was carried out using the GENRES statistical program [79]. The experimental details and analysis of phenotypic data was described previously [48].

### Population structure, LD and marker trait association analysis

#### Population structure and kinship

A total of 204 simple sequence repeat (SSR) markers (S3 Table) were selected from all 12 chromosomes to determine population structure and kinship. Genotyping followed the methodology described by Geethanjali et al. [80]. An admixture model with correlated allele frequency in STRUCTURE 2.2 software [81] was applied with a burn-in of 30,000 iterations and a total Markov chain Monte Carlo (MCMC; [82]), length of 100,000 to test a number of populations (k) with values from 2–10. Each k was replicated five times and the run that assigned more lines with possibility of > 0.5 in all clusters was used. The likely number of sub-populations was determined using the approach of Evanno et al. [50], in which the change of k (delta k) was maximized. The Pearson correlation coefficients were calculated between Q1 value and each phenotypic trait (R^2^) to understand the influence of population structure on salt tolerance.

Kinship matrix (k) was calculated according to Ritland [83] using the SPAGeDi software package [84]. The SSR markers with allele frequency less than 10% and more than 10% missing data were discarded from the analysis. The distribution of pairwise kinship data points was summarized using Excel’s internal function ‘frequency'. Diagonal of the matrix was set to 2 and all negative values between individuals were set to zero [25]. Q matrix from STRUCTURE and k matrix from SPAGeDi were formatted to a text file readable by TASSEL for association analysis.

#### Candidate gene specific primer design, amplification, sequencing, and LD analysis

Reference sequences for the selected candidate genes DREB1A (complete cds, AF00011), NHX1 (partial cds, AJ306630), VP1.1 (partial cds, AJ278019) and TIP (partial cds, AY731066) were obtained from the GenBank database of the National Centre for Biotechnology Information (NCBI; http://www.ncbi.nlm.nih.gov/genbank/). Primer3 tool was used to design primers based on reference sequences for PCR amplification of the selected candidate genes in order to give maximum sequence coverage [85] (Table 7).

**Table 7:** Primer sequences, amplicon coverage, and % identity to reference sequence for the selected candidate genes.

Reference sequence	Forward primer	Reverse primer	Coverage % of the amplicon	Identity % of amplicon
>gi|25992099|gb|AF500011.1| *Lycopersicon esculentum* dehydration responsive element binding protein (DREB1A) mRNA, complete cds	CTCTACAAGCTTTTTGTTAAGAGAC	CCACATCTTAAGATTACCGCAGAAT	100	99
>gi|14149006|emb|AJ278019.1| *Lycopersicon esculentum* partial mRNA for vacuolar-type H^+^-pyrophosphatase (VP1.1 gene)	GACTCATTATTGGGTTTGTC	ACTGATACAATTACTCTAGG	76	99
>gi|15982203|emb|AJ306630.1| *Lycopersicon esculentum* mRNA for Na^+^/H^+^ antiporter (NHX1 gene)	TCCTGGAAAATCTGTTGGGG	TGAATCCCCTTTAGCTCCGC	50	100
>gi|52355223|gb|AY731066.1| *Lycopersicon esculentum* putative aquaporin TIP-type mRNA, partial cds	GCTCAATTACTTGGCTCCA	GTCTTCTGAGGTTGGAA	67	100

Source: GenBank database of the National Centre for Biotechnology Information (http://www.ncbi.nlm.nih.gov/genbank/)

DNA extraction followed the methodology described by Geethanjali et al. [80]. The PCR amplicons were purified using a QIAquick PCR Purification Kit (Qiagen) and sequenced using an ABI 3700 DNA analyzer (Applied Biosystems, USA) (genedragon.com.tw). The nucleotide sequences for DREB1A (KM094061-KM094129), VP1.1 (KM244580-KM244651), NHX1 (KM093902-KM093974) and TIP (KM093975-KM244651) have been deposited in the NCBI Genbank database. To identify SNPs, contigs were first generated by aligning forward and reverse sequences of each genotype using the contig assembly program implemented in Bioedit 7.0.0 [86]. The contig sequences of the 94 genotype panel were used to develop multiple sequence alignments using the Clustal W program implemented in Bioedit. These multiple sequence alignment files were used to extract SNPs and indels with minimum allele frequency (MAF) > 5% using SITES command in TASSEL. The SNPs and indels were then used to estimate diversity and intragenic LD in those candidate genes as implemented in TASSEL 2.1 [59]. The reading frame, position of the trait associated SNP/Indels and their effect on the resulting amino acid sequence was studied using ORF finder software (NCBI) and compared with coding sequence (CDS) of the reference accession.

#### Association analysis

Association analysis was conducted to determine the effects of SNPs in each gene on various salt tolerance traits. A mixed linear model [25] was applied for association analysis that evaluated the effects of SNPs/indels/haplotypes with MAF > 5% individually, adjusting for population structure and kinship as implemented in TASSEL 2.1 [59]. To avoid the problem of multiple testing during association analysis, the False Discovery Rate (FDR) corrected significance values were calculated using QVALUE [26]. The *q*-value is a measure of significance in terms of the false discovery rate similar to the *p*-value that relates to the false positive rate. Marker-trait associations having a *q*-value equal or inferior to 0.05 were declared significant. The P and q values determine whether a locus/haplotype is associated with the marker and the marker R^2^ evaluates the magnitude of the locus effect [87].

## Supporting Information

S1 Table Mean performance of 94 accessions of *S*.*pimpinellifolium* for physiological and yield traits under control and salt stress conditions. (XLS)

S2 Table List of *S*. *pimpinellifolium* accessions used in this study, their country of origin and collection site, where available. Accessions in bold belong to the core set developed by Rao et al. (2011); locations in bold indicate coastal sites.
(DOC)

S3 Table List of 204 SSR markers used for structure and kinship analysis along with their position, repeat motif, and primer sequence. (XLS)
