# Supplementary material for: Variations in DREB1A and VP1.1 Genes Show Association with Salt Tolerance Traits in Wild Tomato (Solanum pimpinellifolium)
Source: PLoS One. 2015 Jul 10;10(7):e0132535. doi: 10.1371/journal.pone.0132535 (PMC4498769; doi:10.1371/journal.pone.0132535)
Supplement: S2 Table — Accessions in bold belong to the core set developed by Rao et al. (2011); locations in bold indicate coastal sites. (DOC) [file pone.0132535.s002.doc]

**Table S2** List of *S. pimpinellifolium* accessions used in this study, their country of origin and collection site, where available. Accessions in bold belong to the core set developed by Rao et al. (2011); locations in bold indicate coastal sites.

| Genotype No. | AVRDC Accession No. | Donor Code | | Country of Origin | Location |
| --- | --- | --- | --- | --- | --- |
| **1** | **VI005553** | LA 1261 SAM 343-1 | | Ecuador | Babahoyo, Los Rios, left bank of the San Pablo River; 10 m |
| **2** | **VI005554** | LA 1263 SAL 345 | | Ecuador | Barranco Chico, Guayas Province; 50 m |
| **3** | **VI005572** | LA 1382 SAL 466 | | Peru | Chachapoyas to Balsas, Amazonas province |
| **4** | **VI005575** | LA 1416 SAL 504 | | Ecuador | Las Delicias, Pichincha Province; 500 m |
| 5 | VI005591 | LA 1466 | | Peru | Chongoyape, Lambayeque Province; 250 m |
| **6** | **VI005797** | PI 79532; LA2348 | | Peru | **Near Trujillo, La Libertad Region; coastal northwestern Peru; 34 m** |
| 7 | VI006035 | PI 126430 | | Peru | Carabayllo District, Lima Province; 238 m |
| **8** | **VI006037** | PI 126432 | | Peru | Carabayllo District, Lima Province; 238 m |
| **9** | **VI006038** | PI 126433 | | Peru | Carabayllo District, Lima Province; 238 m |
| 10 | VI006073 | PI 126925 | | Peru | **Pacasmayo, La Libertad; near Pacific ocean; 8 m** |
| 11 | VI006081 | PI 126933 | | Peru | **Huarmey, Ancash Region, near Pacific ocean; 60 m** |
| 12 | VI006085 | PI 126937 | | Peru | Mache, La Libertad Region, Otuzco Province; 3300 m |
| **13** | **VI006087** | PI 126939 | | Peru | **Santa Province, Ancash Region; near Pacific coast** |
| **14** | **VI006089** | PI 126941 | | Peru | **Casma, Ancash Region, near Pacific coast; 210 m** |
| 15 | VI006095 | PI 126947 | | Peru | **Chan Chan, near Pacific coast; 60 m** |
| **16** | **VI006100** | PI 126952 | | Peru | **Between Zana and Chiclayo, 13 km from Pacific ocean; 46 m** |
| 17 | VI006101 | PI 126953 | | Peru | **Lambayeque, coastal region of Northern Peru; 8 m** |
| **18** | **VI006102** | PI 126954 | | Peru | **Lambayeque, coastal region of Northern Peru; 8 m** |
| 19 | VI006119 | PI 127807 | | Peru | Hacienda Chinche, near Quillabamba, southern Sierra of Peru; 1158 m |
| **20** | **VI006144** | PI 127833 | | Peru | Quillabamba, southern Sierra of Peru; 1158 m |
| **21** | **VI006466** | PI 133542 | | Ecuador | Not available |
| 22 | VI006536 | PI 143527 | | Peru | Not available |
| 23 | VI006934 | PI 205009 | | USA | Sullana, Sullana Province,on the Chira River, northwestern coastal desert of Peru |
| 24 | VI006995 | PI 211840 | | Peru | **Trujillo Province, La Libertad Region; coastal northwestern Peru** |
| **25** | **VI007001** | PI 212408 | | Peru | Rio Supe, Peru, Barranca Province |
| **26** | **VI007002** | PI 212409 | | Venezuela | Not available |
| **27** | **VI007090** | PI 224709 | | Mexico | From a market in San Cristobal, Chiapas, Mexico. Growing along a river bank. |
| **28** | **VI007091** | PI 224710 | | Mexico | Teopisca, Municipality of Chiapas, southern Mexico |
| 29 | VI007097 | PI 230327 | | Ecuador | Tropical Experimental Station of Pichilingue on the Quevedo river |
| **30** | **VI007159** | PI 251315; LA0381 | | Peru | Pongo, La Libertad Province; 300 m |
| **31** | **VI007160** | PI 251316; LA0384 | | Peru | Chilete at Rio Jequetepeque, Cajamarca Province; 500 m |
| **32** | **VI007161** | PI 251317; LA0397 | | Peru | Hacienda Tuman, Lambayeque; 700 m |
| **33** | **VI007162** | PI 251318; LA0400 | | Peru | Hacienda Buenos Aires, Piura Province; 800 m |
| 34 | VI007163 | PI 251319; LA0411 | | Ecuador | Pichilingue, Los Rios Province; 200 m |
| **35** | **VI007164** | PI 251320; LA0412 | | Ecuador | Pichilingue, Los Rios Province; 250 m |
| 36 | VI007167 | - | | Ecuador | Not available |
| **37** | **VI007326** | PI 263589 | | Mexico | **Ahome municipality, coast of the Gulf of California, state of Sinaloa** |
| **38** | **VI007510** | PI 270439 | | Mexico | Papantla, state of Veracruz; 900 m |
| **39** | **VI007514** | PI 270443 | | Mexico | Papantla, state of Veracruz; 900 m |
| 40 | VI007515 | PI 270444 | | Mexico | Papantla, state of Veracruz; 900 m |
| 41 | VI007518 | PI 270447 | | Mexico | Papantla, state of Veracruz; 900 m |
| **42** | **VI007519** | PI 270448 | | Mexico | Municipality of Juárez, state of Chihuahua; 1085 m |
| **43** | **VI007523** | PI 270452 | | Mexico | Municipality of Juárez, state of Chihuahua; 1085 m |
| 44 | VI007524 | PI 270453 | | Mexico | Not available |
| **45** | **VI008092** | PI 274174 | | Mexico | Not available |
| 46 | VI008107 | PI 279373 | | Guatemala | Not available |
| **47** | **VI008705** | PI 313943; LA1993 | | Peru | Chicama Valley? Lima Province |
| **48** | **VI009082** | PI 365906; LA1255 | | Ecuador | Loja, Province of Loja; 2200 m |
| 49 | VI009087 | PI 365914; LA1259 | | Ecuador | Catarma, Los Rios Province; 20 m |
| **50** | **VI009088** | PI 365915; LA1260 | | Ecuador | Pueblo Viejo, Los Rios Province; 10 m. |
| 51 | VI009089 | PI 365916; LA1261 | | Ecuador | Babahoyo, Los Rios Province; 10 m |
| **52** | **VI009091** | PI 365918; LA1263 | | Ecuador | **Barranco Chico, Guayas Province; 50 m** |
| **53** | **VI009109** | PI 365963; LA1349 | | Peru | **Cuculi, Lambayeque Province; 180 m** |
| 54 | VI009110 | PI 365965; LA1357 | | Peru | Jimbe, Ancash Province; 1000 m |
| 55 | VI009111 | PI 365966; LA1359 | | Peru | La Crau, Ancash Province; 1000 m |
| **56** | **VI009550** | PI 407545; LA 1589 | Peru | | Vira - Galunga, lower Vira valley, La Libertad Province |
| 57 | VI009620 | PI 379020; LA1341 | Peru | | Huampani, 3 km east of Nana, Lima Province: 500m |
| **58** | **VI009622** | PI 379022; LA1348 | Peru | | **Pacasmayo, La Libertad Province; 50 m** |
| 59 | VI009771 | PI 390704 | Peru | | **Piura, between Chulucanas and Tambo Grande; 68 m** |
| **60** | **VI010049** | - | Indonesia | | Not available |
| 61 | VI030545 | BL 358 | Mexico | | Not available |
| **62** | **VI030546** | BL 359 | Mexico | | Not available |
| **63** | **VI030547** | BL 360 | Mexico | | Not available |
| **64** | **VI030548** | BL 361 | Mexico | | Not available |
| 65 | VI030392 | BL 203; PI 306216 | Argentina | | Not available |
| 66 | VI037266 | LA 411 | Ecuador | | Pichilingue, Los Rios Province; 200 m |
| 67 | VI037269 | LA 1237 | Ecuador | | **Atacames, Esmeraldas Province; 5 m** |
| 68 | VI037271 | LA 1246 | Ecuador | | La Toma, Loja Province; 1200 m |
| 69 | VI037273 | LA 1279 | Peru | | Cieneguilla, Lima Province; 200 m |
| 70 | VI037278 | LA 1478 | Peru | | Santo Tome (Pabur), Piura Province; 100 m |
| **71** | **VI037279** | LA 1521 | Peru | | **El Pinon, Asia, Lima Province; 150 m** |
| **72** | **VI037280** | LA 1547 | Ecuador | | Chota to El Angel, Carchi Province; 3000 m |
| 73 | VI037283 | LA 1582 | Peru | | Motupe, Lambayeque Province; 164 m |
| 74 | VI037285 | LA 1586 | Peru | | Zana, San Nicolas, La Libertad Province |
| **75** | **VI037290** | LA 1606 | Peru | | **Tambo de Mora, Ica Province; 10 m** |
| 76 | VI037292 | LA 1659 | Peru | | Pariacoto, Ancash Province; 1350 m |
| **77** | **VI037973** | LA 2183 | Peru | | Corral Quemado, Amazonas Province; 450 m |
| 78 | VI037974 | LA 2401 | Peru | | Near Cerro Sechin, Casma valley, Ancash Province; 100 m |
| 79 | VI037975 | LA 2533 | Peru | | Lomas de Latillo, Lima Province; 400 m |
| **80** | **VI037972** | LA 2181 | Peru | | Balsa Huaico, Cajmarca Province; 850 m |
| 81 | VI008942 | PI 346340 | Peru | | Not available |
| 82 | VI009215 | PI 370093 | Canada | | Not available |
| **83** | **VI009543** | LA 1581 | Peru | | Punto Cuatro, Lambayeque Province |
| **84** | **VI009548** | LA 1587 | Peru | | **San Pedro de Lloc, La Libertad Province; 40 m** |
| **85** | **VI009549** | LA 1588 | Peru | | **Laredo to Barraza, La Libertad Province; 50 m** |
| **86** | **VI009628** | PI 379028; LA1384 | Peru | | **QuebradaParca, Lima Province** |
| **87** | **VI009649** | PI 379057 | Ecuador | | From Hacienda Carmela, Guayas Province |
| **88** | **VI009651** | PI 379059; LA1258 | Ecuador | | Voluntario de Dios, Azuay Province |
| **89** | **VI009758** | PI 390689 | Peru | | **Paramonga 28 km on road to Huaraz** |
| **90** | **VI009760** | PI 390693 | Peru | | **Between Trujillo and Chiclayo** |
| **91** | **VI009762** | PI 390695 | Peru | | **Between Trujillo and Chiclayo. Habitat: Collected from salt encrusted soil.** |
| **92** | **VI009763** | PI 390696 | Peru | | **Between Trujillo and Chiclayo** |
| **93** | **VI009765** | PI 390698 | Peru | | **Km 681 on Pan American Highway (north edge of Guadeloupe)** |
| 94 | VI057411 | LA 1579 | Peru | | **Colegio Punto Cuatro #1, Lambayeque Province** |
